# Adult appendicitis score versus Alvarado score: A comparative study in the diagnosis of acute appendicitis

**DOI:** 10.1016/j.sopen.2023.07.007

**Published:** 2023-07-20

**Authors:** Mohamed Said Ghali, Samer Hasan, Omer Al-Yahri, Salah Mansor, Mohannad Al-Tarakji, Munzir Obaid, Amjad Ali Shah, Mona S. Shehata, Rajvir Singh, Raed M. Al-Zoubi, Ahmad Zarour

**Affiliations:** a Department of Surgery, Acute Care Surgery, Hamad Medical Corporation, Doha, Qatar; b Department of General Surgery, Ain Shams University, Cairo, Egypt; c Department of Pharmacy, Women's Wellness and Research center, Hamad Medical Corporation, Doha, Qatar; d Surgical Research Section, Department of Surgery, Hamad Medical Corporation, Doha, Qatar; e Department of Chemistry, Jordan University of Science and Technology, P.O.Box 3030, Irbid 22110, Jordan; f Department of Biomedical Sciences, QU-Health, College of Health Sciences, Qatar University, Doha 2713, Qatar; g Weill Cornell medical college, Doha, Qatar

**Keywords:** Acute appendicitis, Adult appendicitis score, Negative appendicitis, Alvarado score

## Abstract

**Background:**

Acute Appendicitis (AA) is the most common abdominal surgical emergency. It requires proper management to decrease mortality and morbidity. Clinical scoring systems for diagnosing AA aimed to decrease the use of radiological scans and the rate of negative appendectomies (NA). We aim to assess the adult appendicitis score (AAS) in the diagnosis prediction of AA.

**Method:**

A retrospective study with 1303 cases of AA is performed. We compared the correlation of AAS and Alvarado scores to postoperative histopathology. Specificity, sensitivity, Positive Predictive Value (PPV), and Negative Predictive Value (NPV) were assessed. ROC was used.

**Results:**

AAS risk stratification was applied to the study population. Group I for a low probability, and groups II and III for an intermediate and high probability of AA. We found that 159 patients were matched in group I, 505, and 639 were in groups II and III of AAS, respectively. The correlation between Alvarado and AAS with HP was significant. AAS ≥ 16 presented sensitivity and specificity of 50 % and 75.47 %, respectively, with PPV of 97.96 % and NPV of 6.02 %, with an accuracy of 51.04 %. Regarding AAS ≥ 11, the sensitivity was 88.96 %, specificity was 39.62 %, PPV was 97.2 %, NPV was 13.21 %, and accuracy was 86.95 %.

**Conclusion:**

AAS is relatively more accurate than Alvarado's score, especially in selecting a safe candidate for discharge from an emergency. In addition, AAS is found to decrease the need for radiological images and NA rate more than Alvarado.

## Introduction

Acute Appendicitis (AA) is a frequently encountered abdominal surgical emergency with an estimated lifetime risk of 7–8 % [1]. In developed countries, it occurs at a rate of approximately 90–100 cases per 100,000 population per year, affecting adolescents and young adults, with a higher incidence among males [2]. Furthermore, severe cases of AA have been associated with increased mortality. Hence, the diagnosis of AA can pose challenges with various differential diagnoses, especially in females, and any delays in treatment can result in elevated mortality and morbidity rates [3,4].

Our institution's employs a standard diagnostic approach for assessing AA, which depends on both physician clinical assessment and the use of radiological modalities. Diagnosing AA can be challenging, as rely solely on clinical diagnosis carries a significant risk of negative appendectomy (NA), with rates reported in the literature reaching up to 23 % [4,5]. However, incorporating imaging studies into the diagnostic process has been shown to improve the accuracy of AA diagnosis while reducing the rate of NA [5,6]. It is important to note that in cases of typical appendicitis, the use of imaging may potentially lead to a delay in surgical consultation and intervention, therefore increasing the risk of complications [7].

Since the establishment of clinical scoring systems, they have played a remarkable role in improving diagnostic accuracy and reducing the need for further investigations like US, CT scan, and MRI. These scoring systems are based on symptoms, signs, and laboratory findings, helping to raise clinical suspicion of AA without providing a definitive diagnosis. They assist in appropriately selecting patients with uncertain diagnoses for diagnostic imaging.

Alvarado score is widely recognized as the most renowned scoring system for diagnosing AA in adults [8]. Its components include eight factors: migratory pain to the Right iliac fossa, anorexia, nausea, vomiting, temperature >37.3 °C, rebound tenderness, and neutrophilic count >75 %. Each of these factors scored 1. Additionally, tenderness of the Right iliac fossa and leukocytes >10,000/Ul are scored 2. Therefore, the total score is calculated by summing up the scores according to available components, resulting in the 10-point Alvarado score [7]. However, the diagnostic power of the Alvarado score in predicting AA was assessed in a previous study and concluded that it was insufficient to be considered the main scoring system in our institute [9]. As a result, a new scoring system called the adult appendicitis scoring system (AAS) was established by Sammalkorpi et al. in 2014 [10].

According to the updated guidelines from the World Society of Emergency Surgery (WSES), the use of AAS is recommended, while the use of the Alvarado score to help increase clinical suspicion of AA in adults is discouraged [11]. In our institution, many physicians are utilizing AAS in diagnosing AA instead of relying on the Alvarado score. However, there is a lack of sufficient published research on AAS assessment despite the WSES recommendations. Herein, the aim of this study is to assess the effectiveness of AAS compared to Alvarado's score in predicting the diagnosis and stratifying the risk of AA in correlation to HP as the gold standard for diagnosis.

## Materials and methods

### Study population

The study is a secondary data carried out at Hamad Medical Corporation (HMC), Qatar's main health care provider. The study period was from January 1st, 2018, until January 31st, 2019, as approved time frame by the ethical committee for human research by the Medical Research Center of HMC with protocol number (MRC/01/19/454). 1303 patients diagnosed with AA were included in the study. Our inclusion criteria were: (1) All patients ≥14 years old, (2) patients who were admitted with AA and underwent appendectomy, and (3) postoperative histopathology results were available.

### Study data and diagnostic scores

The electronic medical records (EMR) database was used to search for study data. Collected data in this study demonstrate three sets of pre, intra, and postoperative data. The first set included demographic, history, and clinical characteristics. Second set of data demonstrates laboratory results and radiological findings. Finally, the third data set includes surgical procedure details, hospital course and Histopathology (HP) grading.

For comparison, AAS and Alvarado scores were calculated retrospectively using their components, as demonstrated in Table 1, Table 2. Retrospective calculation of these scores is based on history, physical examination, and comprehensive laboratory testing, which are accessible on a regular basis not just for patients with acute appendicitis but also for individuals who report to emergency departments with acute abdominal pain. We stratified them according to the risk of having AA into group I for a low probability of AA, and groups II and III for an intermediate and high probability of AA. The AAS Risk Stratification was generated based on the score result. So, a score of 0–10 represents a low probability, a score of 11–15 is considered intermediate probability, and an AAS score of ≥16 is considered high probability of AA. In terms of Alvarado Risk Stratification, a score of 1–4 indicates low probability, a score of 5–6 indicates intermediate probability, and a score of 7–10 indicates high probability of AA.

**Table 1:** Alvarado score^a^.

Alvarado components	Score points
*Symptoms*	
Migratory Pain to Right Iliac Fossa	1
Anorexia	1
Nausea or Vomiting	1
*Signs*	
Tenderness of The Right Iliac Fossa	2
Rebound Tenderness	1
Temperature >37.3 °C	1
*Laboratory*	
Leukocytes Above 10,000 ^3/Ul	2
Neutrophilic Count >75 %	1

a Alvarado Risk Stratification; Score 1–4 For Low Probability, score 5–6 For Intermediate Probability, Score 7–10 For High Probability of AA.

**Table 2:** Adult appendicitis score (AAS)^a^.

Symptoms and findings		Score
Pain in RLQ		2
Pain relocation		2
RLQ tenderness		3/1^b^
Guarding	Mild	2
	Moderate or severe	4

Laboratory tests		
Blood leukocyte count (×10^9^)	≥7.2 and <10.9	1
	≥10.9 and <14.0	2
	≥14.0	3
The proportion of neutrophils (%)	≥62 and <75	2
	≥75 and <83	3
	≥83	4
CRP (mg/l), symptoms < 24 h	≥4 and <11	2
	≥11 and <25	3
	≥25 and <83	5
	≥83	1
CRP (mg/l), symptoms > 24 h	≥12 and <53	2
	≥53 and <152	2
	≥152	1

**AAS** = Adult Appendicitis Score; **CRP** = serum C reactive protein; **RLQ** = right lower quadrant.

a AAS Risk Stratification; Score 0–10 For Low Probability, score 11–15 For Intermediate Probability, Score ≥ 16 For High Probability Of AA.

b Men and women age 50+/women, age 16–49.

The output of study variables with a comparison between scores is tabulated as shown in Table 3, Table 4. The scores were displayed by ROC analysis, and the area under the ROC curve (AUC) was estimated.

**Table 3:** Demographic, clinical, vitals and laboratory data about Alvarado and AAS risk stratifications.

	Alvarado			AAS			Total (1303)	*P* value	
	Group I (121)	Group II (336)	Group III (846)	Group I (159)	Group II (505)	Group III (639)		Alvarado	ASS
Age (years)	34 ± 10	32 ± 8.8	32 ± 10	32.5 ± 9.2	32.7 ± 9.7	32 ± 9.5	32.3 ± 9.5	0.12	0.52
Hospital stay (days) (IQR)	1.7 ± 0.8	2 ± 1.7	2 ± 2.1	1.7 ± 1.1	2.1 ± 2.5	2 ± 1.6	2 ± 1.96	0.25	0.10
Surgery waiting time (hours)	25.6 ± 12	25.8 ± 13	24 ± 10.5	26 ± 13.3	24.7 ± 11.9	24.1 ± 10.6	24.6 ± 11.5	0.001	0.19
Duration of symptoms (Days)	2.6 ± 2.7	1.9 ± 1.5	1.7 ± 1.2	2.7 ± 2.5	1.8 ± 1.3	1.6 ± 1.2	1.8 ± 1.5	0.001	0.001
BMI	26.3 ± 4.7	25.7 ± 4.7	25.2 ± 4.9	26 ± 5	25.6 ± 4.9	25.1 ± 4.8	25 ± 5	0.02	0.1
SBP (mmHg)	122 ± 14	119 ± 12	120 ± 13	120 ± 13.9	119.5 ± 12.7	119.5 ± 12.5	120 ± 13	0.15	0.9
DBP (mmHg)	74.4 ± 8.9	73 ± 10	72 ± 9	72 ± 9.2	72.4 ± 9.6	72.9 ± 9.4	73 ± 9	0.08	0.5
Pulse rate (beat/min)	77 ± 13	78 ± 12.9	81 ± 13	79.2 ± 12.6	79 ± 13.9	80.5 ± 12.8	80 ± 13	0.003	0.12
Temperature (celsius)	36.8 ± 0.4	36.8 ± 0.5	36.9 ± 0.6	36.8 ± 0.5	39.9 ± 0.6	36.9 ± 0.6	36.9 ± 0.6	0.001	0.07
Oxygen saturation	99 ± 0.9	99 ± 0.8	99 ± 1.2	99 ± 0.8	99 ± 1.2	99 ± 1.2	99 ± 1.1	0.57	1
WBC (^3/uL)	8.2 ± 2.5	11.3 ± 4.3	15 ± 3.6	8.3 ± 2.8	12 ± 3.6	15.7 ± 3.7	13.4 ± 4.3	0.001	0.001
Neutrophils count (^3/uL)	5.3 ± 1.9	8.3 ± 4.1	12.4 ± 3.5	5.2 ± 2.3	9.3 ± 3.4	13 ± 3.6	10.6 ± 4.3	0.001	0.001
Lymphocytes count (^3/uL)	2.1 ± 0.9	2.1 ± 0.9	1.6 ± 0.9	2.2 ± 1	1.9 ± 0.9	1.6 ± 1	1.8 ± 1.0	0.001	0.001
Platelets count (^3/uL)	245 ± 58	251 ± 63	259 ± 64	258.8 ± 64.5	250.7 ± 62.3	258.2 ± 63.1	255 ± 63	0.02	0.1
Hemoglobin level (gm/dL)	14 ± 1.7	14.3 ± 1.9	14.4 ± 1.7	13.7 ± 2.1	14.1 ± 1.7	14.7 ± 1.6	14.3 ± 1.7	0.02	0.001
INR	1.0 ± 0.1	1.2 ± 0.1	1.1 ± 0.2	1.1 ± 0.1	1.1 ± 0.1	1.1 ± 0.2	1.1 ± 0.2	0.09	0.4
Serum creatinine (umol/L)	73.7 ± 16.4	72.5 ± 17.6	73.8 ± 23.9	67.7 ± 18.4	73 ± 27.6	75.3 ± 16.5	73.5 ± 21.8	0.64	0.001
Serum BUN (umol/L)	3.8 ± 1.2	3.9 ± 2.6	4.0 ± 2.7	4 ± 3.5	3.9 ± 2.6	3.9 ± 2.3	3.9 ± 2.6	0.82	0.9
pH	7.8 ± 0.03	7.4 ± 0.04	7.4 ± 0.04	7.4 ± 0.03	7.4 ± 0.1	7.4 ± 0.04	7.4 ± 0.04	0.008	0.2
1Base excess (mmol/L)	1.2 ± 1.6	0.9 ± 1.5	1.0 ± 1.5	0.8 ± 1.9	0.9 ± 1.5	1.1 ± 1.4	1.0 ± 1.5	0.41	0.3
Serum CRP (mg/L)	31 ± 33	42 ± 61	53 ± 75	30.5 ± 49.7	54.4 ± 78	48.5 ± 66.6	49 ± 70	0.03	0.03
Serum lactate	1.6 ± 0.6	1.8 ± 0.8	2.1 ± 0.9	1.6 ± 0.7	1.8 ± 0.8	2.2 ± 0.9	1.9 ± 0.9	0.001	0.001
Serum albumin (gm/L)	39 ± 3.7	38.9 ± 4.4	39.5 ± 4.2	38.8 ± 4.2	38.9 ± 4.2	39.8 ± 4.2	39 ± 4.2	0.34	0.01
Serum glucose (mmol/L)	5.9 ± 2.1	5.8 ± 1.3	6.4 ± 2.1	5.8 ± 1.9	6.1 ± 1.9	6.4 ± 2	6.2 ± 1.9	0.001	0.001
CT scan of appendicular diameter	10.7 ± 2.4	10.5 ± 2.6	11 ± 2.7	10.4 ± 2.8	10.8 ± 2.5	11.6 ± 2.7	11.1 ± 2.7	0.001	0.001

Data are presented as mean ± standard deviation.

**AAS** = Adult Appendicitis Score; **BMI** = Body mass index CT scan = computerized tomography scan; **CRP** = serum C reactive protein; **DBP** = diastolic blood pressure; **IQR** = interquartile range; **INR** = international normalized ratio; **pH** = blood degree of acidity or alkalinity; **SBP** = Systolic blood pressure; **WBCs** = white blood cells.

**Table 4:** Demographic, radiological and clinical characteristics of Alvarado and AAS risk stratifications.

	Alvarado			AAS			Total (1303)	P value	
	Group I (121)	Group II (336)	Group III (846)	Group I (159)	Group II (505)	Group III (639)		Alvarado	AAS
Gender (Male)	89(73.6)	250(74.4)	649(76.7)	88(55.3)	361(71.5)	539(84.4)	988(75.8)	0.58	0.001
Nationality								0.96	0.1
Asian	97(80.2)	268(79.8)	689(81.4)	117(73.6)	405(80.2)	532(83.3)	1054(80.9)		
African	23(19.0)	64(19.0)	149(17.6)	40(25.2)	96(19)	100(15.6)	236(18.1)		
Others	1(0.8)	4(1.2)	8(0.9)	2(1.3)	4(0.8)	7(1.1)	13(1.0)		
Migratory abdominal pain	37 (30.6)	180 (53.6)	651 (77.0)	56(35.2)	293(58)	519(81.2)	868(66.6)	0.001	0.001
Fever	16 (13.2)	45 (13.4)	154 (18.2)	22(13.8)	80(15.8)	113(17.7)	215(16.5)	0.08	0.4
Anorexia	27(22.3)	152(45.6)	583(68.9)	80(50.3)	279(55.2)	403(63.1)	762(58.5)	0.001	0.002
Nausea	37(30.6)	179(53.3)	670(79.2)	90(56.6)	337(66.7)	459(71.8)	886(68)	0.001	0.001
Vomiting	25(20.7)	147(43.8)	581(68.7)	66(41.5)	283(56)	404(63.2)	753(57.8)	0.001	0.001
Change in bowel habits	7(5.8)	26(7.7)	70(8.3)	14(8.8)	44(8.7)	45(7)	103(7.9)	0.63	0.53
Smoking	10(8.3)	29(8.6)	70(8.3)	6(3.8)	47(9.3)	56(8.8)	109(8.4)	0.98	0.08
Alcohol consumption	1(0.8)	2(0.6)	13(1.5)	0(0)	7(1.4)	9(1.4)	16(1.2)	0.28	0.3
DM	7(5.8)	15(4.5)	42(5)	7(4.4)	26(5.1)	31(4.9)	64(4.9)	0.84	0.9
HTN	10(8.3)	7(2.1)	44(5.2)	8(5)	22(4.4)	31(4.9)	61(4.7)	0.10	0.9
CAD	1(0.8)	0(0)	3(0.4)	0(0)	4(0.8)	0(0)	4(0.3)	0.34	0.04
CKD	1(0.8)	0(0)	3(0.4)	1(0.6)	2(0.4)	1(0.2)	4(0.3)	0.34	0.6
AF	0(0)	0(0)	1(0.1)	0(0)	0(0)	1(0.2)	1(0.1)	0.76	0.6
Blood culture	0(0)	6(1.8)	13(1.5)	1(0.6)	7(1.4)	11(1.7)	19(1.5)	0.35	0.6
Surgery (laparoscopy)	115(95)	313(93.2)	743(87.8)	152(95.6)	461(91.6)	558(87.3)	1171(89.9)	0.003	0.003
Conversion	0(0)	3(0.9)	5(0.6)	0(0)	4(0.8)	4(0.6)	8(0.6)	0.55	0.54
Operative complication	1(0.8)	5(1.5)	10(1.2)	0(0)	8(1.6)	8(1.3)	16(1.2)	0.83	0.29
Postoperative drain	0(0)	15(4.5)	38(4.5)	1(0.6)	22(4.4)	30(4.7)	53(4.1)	0.60	0.06
Postoperative imaging	3(2.5)	10(3.0)	37(4.4)	4(2.5)	19(3.8)	27(4.2)	50(3.8)	0.38	0.6
Readmission rate	0(0)	3(0.9)	17(2.0)	1(0.6)	3(0.6)	16(2.5)	20(1.5)	0.13	0.02
Reoperation rate	0(0)	0(0)	3(0.4)	0(0)	1(0.2)	2(0.3)	3(0.2)	0.44	0.75
Mortality rate	0(0)	0(0)	1(0.1)	0(0)	1(0.2)	0(0)	1(0.1)	0.76	0.45
WBCs (10000–15,000 ^3/Ul)	75(62)	186(55.4)	309(36.6)	93(58.5)	231(45.7)	246(38.6)	570(43.5)	0.001	0.001
WBCs (>15,000 ^3/Ul)	28(23.1)	80(23.8)	324(38.3)	31(19.5)	154(30.5)	247(38.7)	432(33.2)	0.001	0.001

Data are presented as n (%).

**AAS** = Adult Appendicitis Score; **DM** = Diabetes mellitus; **HTN** = Hypertension; **CAD** = Coronary Artery Disease; **CKD** = Chronic Kidney Disease; **AF** = Atrial fibrillation; **WBCs** = white blood cells.

### Study outcomes

The primary outcome is to validate the diagnostic accuracy of AAS in diagnosing AA. The secondary outcomes are the ability of AAS to reduce the use of clinical imaging studies in diagnosing AA and the diagnostic role of Alvarado score in comparison with AAS.

### Grading setting

HP findings, we classified the microscopic finding to grade 0 for normal or no evidence of AA, grade I for a mild form of AA, grade II referred to gangrenous/perforated AA, and grade III for AA with incidental neoplastic finding. Regarding Operative findings; it was described as grade 0 for the normal appearance of the appendix, grade I for nonperforated AA, grade II for gangrenous /impending perforation AA, grade III for perforated AA with the collection, grade IV for mass forming AA and grade V as finding mentioned before with superadded generalized peritoneal contamination. We designed this grade description according to The American Association for the Surgery of Trauma (AAST) [12] grading for AA as it is the nearest one to our reported findings. We compare the correlation of Alvarado score and AAS to the gold standard HP findings and intraoperative findings. We chose the cut-off point of Alvarado score at five, seven and eleven; on the other side, we took scores eleven, sixteen and eighteen as cut-off points for AAS to assess the Sensitivity, the Specificity, the Positive Predictive Value and the Negative Predictive Value based on ROC curve and previous publication. Sammalkorpi et al. who created AAH score recommended these cut off value (at score 11,16,18) based on statistical analysis and through ROC curve [10]. Regarding Alvarado cut off value along with many articles cut off value of 5, 7, 9, there is systematic review of 42 studies they recommended these cut off values [7,9,13].

### Statistical methods

Descriptive statistics in mean and standard deviation for interval variables and frequency with percentages for categorical variables were calculated according to Alvarado and AAS groups. Chi-square tests were applied to see the association between HP and clinical scoring systems. One-way ANOVAs were performed to see mean differences among HP and both scores groups for all interval variables. ROC curve and c- statistics were performed to see the best discriminate AA disease at a different cut-off value of Alvarado. A *p*-value of 0.05 (two-tailed) was considered a statistically significant level. SPSS 28.0 statistical package was used for the analysis.

## Results

### Study participants

We enrolled 1303 patients who fulfilled the inclusion criteria. The mean age was 32.3 ± 9.5 years, with male predominance (75.8 %). In addition, 81 % of the study's nationality was of Asian origin. Risk stratification groups of Alvarado score were applied to this study cohort; accordingly, group I displayed 121 patients, 336 patients for group II and 846 patients for high probability group III. Regarding AAS stratification of the study population, we found that 159 patients were matched in group I, 505 and 639 patients were in groups II and III, respectively. After examining the correlation of study variables, such as demographics, clinical data, laboratory, and radiological results, we discovered significant differences between the Alvarado and AAS groups within the same scoring system. We found statistical significance between both the Alvarado and AAS groups regarding specific study data, including the duration of symptoms, migratory abdominal pain, anorexia, nausea, vomiting, WBC count, neutrophil count, lymphocyte count, hemoglobin level, serum C-reactive protein (CRP), serum lactate, serum glucose, Computerized Tomography (CT) scan findings of appendicular diameter, and the surgical approach.

. Also, we found that BMI, fever, pulse rate, platelets count, blood degree of acidity or alkalinity (Ph) and surgery waiting time were statistically significant to only Alvarado risk stratification groups. Conversely, AAS risk groups were significantly related to gender (male), nationality, serum creatinine, and readmission, as displayed in Table 3, Table 4.

CT scans were done for 84 % of patients and the diagnostic power of CT was 96.1 %. 13.5 % of patients had ultrasounds before surgery and 2.5 % had surgery without imaging. The duration of symptoms of acute appendicitis was 1.8 days and the mean surgery waiting time was 26.6 h. The mean Alvarado score for the whole cohort was 7 and for AAS was 15. The main surgical procedure was laparoscopic appendectomy (89.9 %) and the conversion rate from laparoscopic approach to open was 0.6 %.

### Operative outcomes

The intraoperative finding was going with normal-looking appendix (grade 0) in 1.5 % of patients. The operative complications rate was (1.2 %) and reoperation was 0.2 % encountered in 3 patients; one patient operated for postoperative abdominal collection not amenable to non-operative management and the other two patients encountered postoperative bleeding that required surgical control. The mean length of hospital stay was two days. The readmission rate was in 20 patients and the main reason was related to abdominal collection discovered in 14 patients. The recorded mortality was only one case (0.1 %). The correlation between Alvarado and AAS on one side and intraoperative findings on the other was significant (*P* = 0.001). However, grade 0 of intraoperative finding was higher in AAS group 1 with less probability which gives more efficiency of AAS to exclude cases from surgical management (2.5 % for Alvarado versus 4.4 % for AAS) see Table 5.

**Table 5:** Intraoperative grade about AAS.

	Alvarado			AAS			Total	P value	
	Group I (121)	Group II (336)	Group III (846)	Group I (159)	Group II (505)	Group III (639)		Alvarado	AAS
Grade 0	3 (2.5)	11 (3.3)	5 (0.6)	7(4.4)	10(2)	2(0.3)	19 (1.5)	0.001	0.001
Grade I	100 (82.6)	274 (81.6)	628 (74.2)	133(83.6)	401(79.4)	468(73.2)	1002 (77)		
Grade II	7 (5.8)	27 (8.0)	149 (17.6)	8(5)	60(11.9)	115(18)	183 (14)		
Grade III	0 (0)	1 (0.3)	4 (0.5)	0(0)	3(0.6)	2(0.3)	5 (0.4)		
Grade IV	11 (9.1)	23 (6.8)	60 (7.1)	11(6.9)	31(6.1)	52(8.1)	94 (7.2)		
Total	121 (100)	336 (100)	846 (100)	159(100)	505(100)	639(100)	1303 (100)		

Data are presented as n (%). **AAS** = Adult Appendicitis Score.

### Correlation between the histopathological findings and the diagnostic scores

Regarding HP findings, there were 52 patients (grade 0) with a normal appendix representing a negative appendectomy rate of 4 %. There is a statistical significance between HP and Alvarado's scoring system and AAS's (*p* = 0.001). However, as mentioned previously, the number of patients in group 1 with grade 0 representing negative appendicitis is more in AAS (21 patients) than in Alvarado (12 patients) which gives another clue of the efficacy of AAS in detecting patients not required surgery as a management option as demonstrated in Table 6.

**Table 6:** HP findings in association with AAS.

HP	Alvarado score			AAS			Total	P value	
	Group I (121)	Group II (336)	Group III (846)	Group I (159)	Group II (505)	Group III (639)		Alvarado	AAS
Grade 0	12 (9.9)	25 (7.4)	15 (1.8)	21(13.2)	19(3.8)	12(1.9)	52 (4)	0.001	0.001
Grade I	108 (88.4)	299 (89)	791 (93.6)	133(83.6)	469(92.9)	596(93.3)	1198 (92)		
Grade II	1 (0.8)	8 (2.4)	33 (3.9)	1(0.6)	14(2.8)	27(4.2)	42 (3.2)		
Grade III	1 (0.8)	4 (1.2)	6 (7.1)	4(2.5)	3(0.6)	4(0.6)	11 (0.8)		
Total	122 (100)	336 (100)	845 (100)	159(100)	505(100)	639(100)	1303 (100)		

Data are presented as n (%). **HP** = Histopathology result; **AAS** = Adult Appendicitis Score.

### ROC curve

**Fig. 1:**
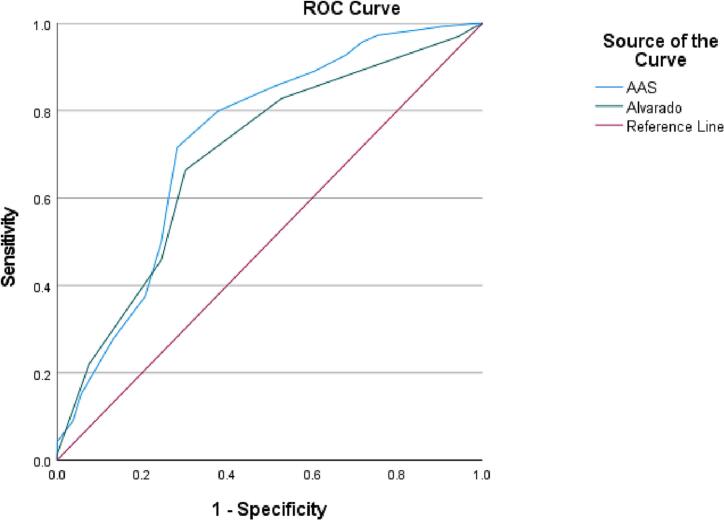
ROC Curve showing a comparison of the new Adult Appendicitis Score (AUC 0.731) compared with Alvarado score (AUC 0.696). AAS = Adult Appendicitis Score; ROC = Receiver operating characteristic.

We utilized (ROC) curve to get the best cut-off values for AAS and Alvarado, which displayed an area under the curve (AUC) of 0.731 and 0.696 for AAS and Alvarado respectively seen in Fig. 1. We computed the sensitivity, specificity, positive predictive value (PPV) and negative predictive value (NPV) with the optimal cut-off value for both scores, as demonstrated in Table 7.

**Table 7:** Sensitivity, specificity, PPV, NPV and accuracy according to different cut-off values of Alvarado and AAS scores.

Cut off	Sensitivity	Specificity	PPV	NPV	Accuracy
Alvarado					
5	91.2 %	22.6 %	96.5 %	9.8 %	88.4 %
7	66.4 %	69.8 %	98.1 %	8.1 %	66.5 %
9	22 %	92.45 %	98.57 %	4.79 %	24.87 %

AAS					
11	88.96 %	39.62 %	97.20 %	13.21 %	86.95 %
16	50.00 %	75.47 %	97.96 %	6.02 %	51.04 %
18	27.76 %	86.79 %	98.02 %	4.85 %	30.16 %

**PPV** = positive predictive value; **NPP** = negative predictive value; **AAS** = Adult Appendicitis Score.

## Discussion

The AA is a commonly encountered surgical emergency worldwide that surgeons manage on a daily basis. It requires an excellent surgical attention because its symptoms can resemble those of other abdominal conditions, especially in females. While AA can present with nonspecific clinical data, it can also manifest in a severe form that poses a significant risk of complications and can have a detrimental impact on a patient's life [14,15]. Surgeons prefer to intervene promptly and proceed with an appendectomy instead of waiting to avoid the risk of complications. However, this approach has led to an increase in negative appendectomies (NA) and unnecessary surgeries. Consequently, there has been a growing need to search and develop a viable diagnostic score that enables accurate diagnosis, while reducing the rate of NA and over-reliance on radiological assessments [16].

In our study, depending on HP results, the NA rate was found to be 4 %, which is favorable compared to the literature rate [[17], [18], [19], [20]]. Surprisingly, we observed no significant difference in the NA rate between the female and male genders (51.9 % and 58.1 % respectively), contradicting previous studies that reported a higher NA rate in female [21,22]. CT scans were performed in 84 % of our cases, with a diagnosis accuracy of 96.1 %. This accuracy is comparable to the literature's reported range of 93 and 98 % [23]. Notably, a separate prospective study demonstrated a high diagnostic accuracy of 97.8 % for CT scans in detecting AA. Interestingly, only 32 % of patients in that study had CT scans performed in the emergency unit [24].

In our study, the readmission rate was 1.5 % (20 patients), with the main reason being postoperative abdominal collection, accounting for (70 %) of the readmissions. This is in contrast to another study which reported a higher readmission rate of 11.9 % among patients, of which 25 % were due to postoperative abdominal collection. Moreover, the other study reported a reoperation rate of 2.5 %, which is clinically significant compared to our study where it was only 0.2 % [25]. A recent meta-analysis reported by Bailey et al. showed a readmission rate of 4.5 % [26]. However, the main causes of readmission were reported as postoperative abdominal collection and pain [27].

We found a significant correlation between both Alvarado and AAS with the following parameters: WBCs count; neutrophils count, lymphocytes count, hemoglobin level, serum C reactive protein (CRP), and serum lactate. These parameters showed an increase in severity grading of AA and were previously considered as biomarkers for diagnosing AA. However, clinically, no single biomarker has demonstrated significant diagnostic performance to be used in isolation [28].

Many scoring systems have been introduced over time. One of the earliest and most commonly used worldwide was the Alvarado score. Numerous studies have been conducted in the literature to validate the Alvarado score for diagnosing AA, yielding mixed results with supporting and non-supporting findings. A recent investigation of the Alvarado score was carried out in our institution, but the study concluded with unsatisfactory Alvarado sensitivity for diagnosing AA [9]. Therefore, we endeavored to validate a new scoring system in our institution, aiming to identify a more suitable score that could accurately diagnosis AA and reduce excessive use of radiological methods. We decided to compare Alvarado score with a recently introduced score called AAS, which was developed in 2014 and is recommended by WSES updates [10]. The confirmatory postoperative histopathology was utilized as the gold standard for AA diagnosis.

We selected a cut-off value according to a previous study, as demonstrated by Chae et al. [29]. Accordingly, the ROC curve we generated as shown in Table 7, revealed that AAS outperforms the Alvarado in terms of area under the curve (AUC) as seen in Fig. 1. AAS exhibited relatively higher accuracy for both higher and lower cut-off values, indicating its superior ability to stratify patients with AA. This finding is consistent with the earlier publication on AAS construction, which strongly supports our results [10]. Furthermore, Kabir et al. [25] reported similar, with AAS having a better AUC (0.78) compared to the Alvarado score (0.75), providing further evidence that AAS can decrease NA and the need of radiological diagnosis. Conversely, Capoglu et al. demonstrated no significant difference in AUC between AAS and Alvarado, along with similar accuracy [30].

After stratifying the AA cases based on HP and intraoperative findings, we noticed that group I of AAS had a higher proportion of normal looking appendices or absence of histological inflammation compared to group I of Alvarado. This suggests that AAS can effectively identify more patients with negative appendicitis and can change of way of management from operative to conservative management, especially in those with lower probability score (group I). Additionally, we observed that a majority of the patients (65 %) fell into high probability group III, according to Alvarado, which is considerably higher than in AAS (49 %). However, despite that, there was no significant change in accuracy. This finding was also reported by Sammalkorpi et al. further supporting its insignificance [10].

After evaluating the sensitivity and specificity of AAS and Alvarado at different cut-off points, we noticed that both scores demonstrated moderate overall diagnostic accuracy, with AAS relatively better performance. Chae et al. also reported similar finding and noted that both scores have been useful in excluding appendicitis in low-risk group I, allowing for safe discharge [29]. Similarly, a recent systematic literature review on the diagnostic value of different scoring systems confirmed that AAS and Alvarado were primarily effective in ruling out appendicitis and identifying low-risk patients for AA, thus reducing the need for radiological evaluations, and minimizing NA rates within these patient groups [31].

Despite the advantages of retrospective medical record reviews, they have inherent limitations regarding data quality. Furthermore, estimating AAS and Alvarado scores retrospectively based on clinical evaluations, which impacted the accuracy of the NA rate. Additionally, variations in the degree of expertise and experience among the operating surgeons may have influenced the recorded intraoperative findings. These limitations should be considered and addressed in future research. Nevertheless, this study has a strength in its large sample size, which provides a more accurate understanding of the relationships between variables. To our knowledge, this is the first study in Qatar to evaluate the correlation between Alvarado score and AAS findings, incorporating a wide range of interrelated variables in such a large sample.

## Conclusion

The diagnosis of acute appendicitis remains a challenging task without radiological confirmation. AAS has demonstrated higher accuracy compared to the Alvarado score, especially in identifying patients suitable for discharge from the emergency department, as it can effectively detect more cases of NA. These findings suggest that AAS reduces the reliance on radiological imaging and decreases the rate of NA more effectively than Alvarado. Therefore, conducting a prospective study is recommended to validate these findings in the near future.

## Implications and contribution

Research working on assessing the adult appendicitis score (AAS) in the diagnosis prediction of AA should consider many of the factors highlighted in the study.

## Funding

The publication of this article was funded by the Qatar National Library.

## Ethical approval statement

This study received approval from the Medical Research Center and Institutional Review Board (IRB) of Hamad Medical Corporation (HMC) on protocol number MRC/01/19/454.

## CRediT authorship contribution statement

**Mohamed S. Ghali:** Conceptualization, Methodology, Investigation, Data Curation, Writing - Original Draft, Writing - Review & Editing. **Samer Hasan:** Methodology, Investigation, Data Curation, Writing - Original Draft. **Salah Mansor:** Methodology, Writing - Review & Editing. **Mohannad Al-Tarakji:** Methodology, Writing - Review & Editing. **Munzir Obaid:** Methodology, Writing - Review & Editing. **Amjad Ali Shah:** Methodology, Writing - Review & Editing. **Mona S. Shehata:** Methodology, Writing - Review & Editing. **Rajvir Singh:** Conceptualization, Methodology and Statistics. **Raed M. Al-Zoubi:** Methodology, Writing - Review & Editing the Final Draft. **Ahmad Zarour:** Conceptualization, Methodology, Writing – final Review & Editing.

## Declaration of competing interest

The authors declare that they have no known competing financial interests or personal relationships that could have appeared to influence the work reported in this paper.

## Data Availability

Data will be made available on request.
